# Supplementary material for: FastqPuri: high-performance preprocessing of RNA-seq data
Source: BMC Bioinformatics. 2019 May 3;20:226. doi: 10.1186/s12859-019-2799-0 (PMC6500068; doi:10.1186/s12859-019-2799-0)
Supplement: Supplementary file 2 — Archive of FastqPuri. Archive containing all files needed to install and run FastqPuri v1.0.6. Date stamp March 22, 2019. (GZ 47,819 kb) [file 12859_2019_2799_MOESM2_ESM.gz › FastqPuri-1.0.6/html/md_README.html]

My Project: FastqPuri, an fq quality control and filter tool


My Project

FastqPuri, an fq quality control and filter tool

Software and source code of `FastqPuri`. It creates quality reports of `fastq` files and filters them removing low quality reads, reads containing too many N's or contamination reads (unwanted rRNA reads, impurities coming from another organism, ...).

## Installation

Clone the repository, or download the source. Make sure that your system supplies the following dependencies for FastqPuri.

- OS: Linux (clang, gcc), Mac OS (clang, gcc), OpenBSD (clang)
- `cmake` (at least version 2.8),
- a `C` compiler supporting the `c11` standard (change the compiler flags otherwise),
- pandoc (optional, see documentation in `PANDOC.md`),
- `Rscript` (optional),
- Following `R` packages installed (optional):
  - `pheatmap`
  - `knitr`
  - `rmarkdown`

**NOTE:** FastqPuri will work without the optional dependencies but will skip creating html reports if they are not available.

$ cmake -H. -Bbuild/ [-DRSCRIPT=myRscriptexec ... ]

$ cd build

$ make

When running cmake, there are some variables you can set using the option -D followed by the variable name. This variables are,

- `CMAKE_C_COMPILER`: `C` compiler (default `gcc`)
- `CMAKE_C_FLAGS`: compiler flags (default `-Wall -O3 -march=native -std=c11`).
- `PANDOC`: `pandoc` executable (default `pandoc`),
- `RSCRIPT`: `Rscript` executable (default `Rscript`),
- `READ_MAXLEN`: Maximum Illumina read length (default 400),
- `RMD_QUALITY_REPORT`: path to `quality_report.Rmd`,
- `RMD_SUMMARY_REPORT`: path to `summary_report.Rmd`,

The executables will be created in the folder `bin`.

**WARNING:** do not move the executables that depend on `R` scripts, anywhere else, unless you also move the corresponding `R` scripts respecting the local folder structure.

## Executables

- `Qreport`: creates a quality report in html format (see `README_Qreport.md`),
- `Sreport`: creates a summary report in html format on a set of samples, regarding either the original files or the filtering process (see `README_Sreport.md`),
- `makeBloom`: creates a bloom filter from a fata file of a certain size, and stores it in a file (see `README_makeBloom.md`)
- `makeTree`: creates a tree of a certain depth from a fasta file and stores it in a file (see `README_makeTree.md`),
- `trimFilter`: performs the filtering process for single-end data (see `README_trimFilter.md`).
- `trimFilterDS`: performs the filtering process for double stranded data (see `README_trimFilterDS.md`).

An exemplar work flow could be:

- `Qreport`
- `Sreport`
- `makeBloom`
- `trimFilter` or `trimFilterDS`
- `Qreport`
- `Sreport`

## Documentation of the code

A Doxygen documentation of the code is available:

- `html` version under the folder `html` (open `index.html` with a browser).
- `pdf` version: `latex/refman.pdf`

## Contributors

Paula Pérez Rubio

## License

GPL v3 (see LICENSE.txt)


---

Generated by  

 1.8.14
